# The prevalence of dental caries and associated factors among students living with disability in the Amhara region, Ethiopia

**DOI:** 10.1002/cre2.646

**Published:** 2022-08-15

**Authors:** Amare T. Tefera, Biruk Girma, Aynishet Adane, Abebe Muche, Tadesse A. Ayele, Kefyalew A. Getahun, Zelallem Aniley, Semira Ali, Simegnew Handebo

**Affiliations:** ^1^ Department of Dentistry, School of Medicine, College of Medicine and Health Sciences University of Gondar Gondar Ethiopia; ^2^ Department of Internal Medicine, School of Medicine, College of Medicine and Health Sciences University of Gondar Gondar Ethiopia; ^3^ Department of Anatomy, School of Medicine, College of Medicine and Health Sciences University of Gondar Gondar Ethiopia; ^4^ Department of Biostatics and Epidemiology, Institute of Public health, College of Medicine and Health Sciences University of Gondar Gondar Ethiopia; ^5^ Department of Pharmacology, School of Pharmacy, College of Medicine and Health Sciences University of Gondar Gondar Ethiopia; ^6^ Department of Special Need and Inclusive Education, College of Education University of Gondar Gondar Ethiopia; ^7^ School of Public Health, St. Paul's Hospital Millennium Medical College Addis Ababa Ethiopia

**Keywords:** dental caries, DMFT, disability, oral health status, special need school students

## Abstract

**Objectives:**

People living with disability are more vulnerable to dental caries and have a high decayed, missed, and filled permanent teeth (DMFT) index and untreated dental disease than nondisabled individuals. In Ethiopia, there is a dearth of information on the oral health status of the disabled population. Hence, this study aimed to determine the prevalence of dental caries and its predictors among special needs school students in the Amhara region, Ethiopia.

**Methods:**

An institution‐based cross‐sectional study was done on special needs school students in the Amhara region from November 2020 to April 2021. The study participants were recruited using a simple random sampling technique using a computer random generator. Data collection was done using the World Health Organization oral health survey tool. Data entry was done using Epi‐data 4.6 and analyzed using SPSS 26. A logistic regression model was used to identify the possible predictors of dental caries.

**Results:**

Four hundred and forty‐three students with a mean age of 15.8 ± 3.8 were included in the study. The prevalence of dental caries was 41.5% (95% confidence interval [CI]: 36.3, 46.0) in permanent dentition with a mean DMFT score of 1.3 ± 1.6. The prevalence of dental caries in primary dentition was 23.1% (95% CI: 11.9, 32.1) with a mean decayed, missed, and filled primary teeth (dmft) score of 1.9 ± 0.2.

Being 7–12 years old (adjusted odds ratios [AOR] = 3.6, 95% CI: 1.6, 8.3), lower grade level (AOR = 2.4, 95% CI:1.3,4.4), poor oral hygiene status (AOR = 2.5, 95% CI: 1.3, 4.8), and lack of parental support during tooth brushing (AOR = 2.2, 95% CI: 1.2, 4.1) were independent predictors of dental caries.

**Conclusions:**

A significant amount of special needs school students in the study area had dental caries. Age, grade level, oral hygiene status, and lack of parent support during tooth brushing were independent predictors of dental caries.

## 1 INTRODUCTION

Disability is an impairment of body function, restriction from social participation, and limitation of daily activity (CDC, 2020). According to the World Health Organization (WHO), 15% of the world population is believed to have some type of impairment (IBGE, 2000), and 80% of them are from developing countries (Hosseinpoor et al., 2013). In Ethiopia, 14.4 million people are living with disabilities, and visual impairment and lower locomotor disability were the most commonly reported disability types in Northwest Ethiopia (Chala et al., 2017; Fitaw & Boersma, 2006).

Dental caries is the most common oral health problem among school‐aged children. A high prevalence of dental caries has been documented in Latin America, Middle East, and South Asian countries (Bagramian et al., 2009; Petersen et al., 2005). However, the prevalence has decreased in developed countries due to improved dental health care, available fluoride products, improved oral hygiene, and higher awareness concerning caries occurrence (Frazão, 2012; Mehta, 2012). Western and Northern European countries have recorded a decrease in caries, but in the countries of Eastern and Central, Europe caries has remained a public health problem (Marthaler, 2004; WHO, 2008).

People living with disability have a higher prevalence of dental caries and decayed, missed, and filled permanent teeth (DMFT) (Lewis et al., 2005; Reid et al., 2003) than the general population (Dos Santos & Nogueira, 2005; Jaber, 2011; Liu et al., 2014). However, Anders and Davis reported that the caries rate in people with intellectual disability (ID) is the same as or lower than the general population. Instead, the rates of untreated caries are higher in people with ID (Anders & Davis, 2010). Likewise, Wilson et al. (2019) reported that people living with ID had poor oral health and more caries compared with the general population (Wilson et al., 2019). This group of disadvantaged populations is more vulnerable to dental caries if they spend most of their time at home and frequently consume cariogenic and unhealthy foods (White et al., 1998). The prevalence of dental caries among the disabled populations was 45% in the United States (Anderson, 2002), 86.4% in Turkey (Altun et al., 2010), 38% in India (Mehta et al., 2015), 51% in Pakistan (Azfar et al., 2008), and 42.4% in Rwanda (Uwayezu et al., 2020).

This group of disadvantaged populations is more vulnerable to dental caries if they spend most of their time at home and frequently consume cariogenic and unhealthy foods.

Oral health care has been recognized as the most common public health problem and unmet need among the disabled population (Rocha et al., 2015). Untreated dental caries has a substantial impact on an individual's quality of life by causing pain, discomfort, trouble chewing, and interfering with everyday activities, such as learning, sleeping, and behavior (Murthy et al., 2014). Moreover, children's nutrition, growth, weight, and overall health are jeopardized due to the severity of decayed teeth (Monse et al., 2010).

In developing countries, oral health is given less attention and the few available oral health services are concentrated in urban areas (Northridge et al., 2020). Furthermore, there is a scarcity of dental professionals in Africa, Asia, and Latin America, where the dental treatments offered are mostly for pain relief and emergency care management (Northridge et al., 2020). Children with disability have been allocated to the same standard of health care as the nondisabled population. However, they faced barriers to practicing their human rights because of the environment they live in rather than their impairment. The additional burden placed on families deepens the impact of economic poverty and may further perpetuate discriminatory attitudes toward these groups (Dowling et al., 2005).

Knowing the dental caries status of students living with disability and its predictors has utmost importance to design interventions. In Ethiopia, studies were conducted on the oral health status of nondisabled school children (Ayele et al., 2013; Tefera et al., 2021; Teshome et al., 2020); however, to the best of our knowledge, there is no data on the dental caries status of students living with disability in Ethiopia. Therefore, this study aimed to determine the magnitude of dental caries and their predictors among students living with disability in the Amhara region, Ethiopia.

## 2 METHODS AND MATERIALS

### 2.1 Study area

The study was conducted in Northwest Ethiopia. All special need schools in northwest Ethiopia were the study sites. In the study area, there are eight special needs schools located in Gondar, Bahir Dar, Debre Markos, and Dessie town. In this region, 696 disabled children attend special needs education; 341 of them are hearing impaired, 129 blind, and 226 intellectually retarded. The total population of the study area was 1,923,888 according to the 2012 Ethiopian central statistical agency, (2012).

### 2.2 Study population and design

An institutional‐based cross‐sectional study was conducted between November 2020 and April 2021 in special needs schools of the Amhara region, Northwest Ethiopia. Students with disability were eligible to participate in the study if they were attending special needs schools and had parental consent and individual assent. Students with emergency medical conditions and who were unable to cooperate during the clinical examination were excluded from the study.

### 2.3 Sample size determination and sampling techniques

The single population proportion formula was used to determine the sample size. A 50% prevalence rate (no previous study in Ethiopia), 95% confidence interval, 5% margin of error, and 15% nonresponse rate were assumed to calculate the sample size. The total number of participants in the study was 443. To enroll study participants, a simple random sample approach employing a computer random generator was used.

### 2.4 Operational definitions

#### 2.4.1 Disability

A disability is any condition of the body or minds (impairment) that makes it more difficult for the person with the condition to do certain activities (activity limitation) and interact with the world around them (participation restrictions).

#### 2.4.2 Dental caries

A tooth was considered decayed when there was frank carious cavitation on any surface of the tooth.

#### 2.4.3 Special need schools

These schools give programs for children who have challenges or disabilities that interfere with learning. Moreover, they provide the support that has not normally provided in general education programs. These schools and programs tailor learning to address each child's unique combination of needs.

## 3 STUDY VARIABLES

### 3.1 Explanatory variables

#### 3.1.1 Sociodemographic variables

The sociodemographic variables included in the questionnaires were the age of the participants, sex, family educational status, educational level of the participants, residency, family monthly incomes, and type of disability.

#### 3.1.2 Oral hygiene practice variables

The section on oral hygiene practice requested the study participants about their oral hygiene. All the participants were categorized as either having a tooth brushing habit or not having a tooth brushing habit. Also, participants with tooth brushing habits have been asked about the frequency of tooth bruising. Moreover, parents' support during tooth brushing was assessed and categorized as assisted tooth brushing or not assisted.

#### 3.1.3 Dietary habits

The section or dietary habit assessed the refined carbohydrate intake of the study participants and all the study participants were categorized as “yes” for those who take carbohydrate foods or “no” for those who were not taking carbohydrate foods.

#### 3.1.4 Oral hygiene status

The oral hygiene status of students with disability was evaluated using the simplified oral hygiene index (OHIS). The OHIS has two components: calculus index simplified and debris index‐simplified, and individuals' OHIS has to be recorded as good (0–1.2), fair (1.3–3.0), and poor (3.1–6.0) (Greene & Vermillion, 1964).

### 3.2 Outcome variables

#### 3.2.1 Dental caries

Dental caries status was assessed by the dental professionals, and the presence of dental caries was assessed using the DMFT index (World Health Organization, 2013). The mean DMFT scores were calculated by adding the decayed, missed, and filled scores, and caries was considered to be present if the mean DMFT> 0.

#### 3.2.2 Data collection tools and procedures

Data collection was done through face‐to‐face interviews and intraoral examination.

#### 3.2.3 Questionnaires and interviews

A pretested structured interview administered questionnaire adapted from the WHO oral health survey tool was implemented (WHO, 2013). The questionnaire was prepared in English and translated from English into the local language, Amharic, using forward and backward translation and it was again translated back into English by another translator to check if the meaning remains the same and if any discrepancies, were corrected before use in the study. The collected data had five parts: sociodemographic characteristics, dietary habits, tooth brushing habits, medical condition, and type of disability. The data were collected using face‐to‐face interviews between the data collectors and study participants. Parents were also involved in the interview due to the assumption that individuals living with some type of disability would have some recall problems about their previous history, and also due to ethical issues.

Data were collected under strict supervision by dental surgeons and special needs experts. The data collectors received a 5‐day training on the study's objectives, research ethics, approach to the interviewee, data collection tools and techniques, and confidentiality during study selection of study participants and data collection. The data collectors wrote all answers to the questionnaires. The supervisor had onsite supervision during the whole data collection period and checked the data daily to ensure its completeness and consistency.

#### 3.2.4 Dental examination

The intraoral examination was evaluated by qualified dental surgeons according to the methods and principles endorsed by WHO criteria (WHO, 2013). The clinical examiners were calibrated and standardized via a series of training, pretest, and discussions of topics and questions that arose throughout the examination period. The clinical examination was conducted in schools using normal light, students' chairs, and mouth mirrors. Participants in wheelchairs with significant physical disabilities were evaluated in their wheelchairs. The dental caries prevalence was assessed on 20 teeth of the primary dentition, and 28 teeth in the permanent dentition. Moreover, to record dental caries experience, the DMFT and decayed, missed, and filled primary teeth (dmft) scores were recorded according to the WHO criteria. Since it was the time of the corona virus disease 2019 (COVID‐19) pandemic, the data collectors implemented maximum infection prevention mechanisms to prevent the cross‐infection of the disease.

### 3.3 Data analysis

The variables were coded, entered into Epi‐data (version 4.6), and transferred to Statistical Package for Social Science (SPSS 26.0; SPSS) for analysis. Descriptive statistics were computed and reported by means and standard deviations for continuous variables, and frequencies and percentages for categorical variables.

Bivariable logistic regression analysis was done to determine the association between the predictor variables and outcome variable (dental caries) using the *χ* ^2^ test. Variables associated with dental caries in the bivariable logistic regression model (*p* < .25) were entered into the multivariable logistic regression model to control confounding variables. The adjusted odds ratio and its 95% confidence interval along its *p*‐values were calculated. The significance level of *p* ≤ .05 were considered for all analysis.

### 3.4 Ethical considerations

The study tool was reviewed and approved by the University of Gondar institutional review board (V/P/RCS/05/541/2020). Furthermore, study permission was obtained from all city educational bureaus officers and directors of the selected schools before the commencement of the study. The study was conducted according to the protocol submitted to the ethical review board. To guarantee data confidentiality, study participants were identified using codes, and unauthorized individuals would not have to access the collected data. All study participants, parents, or legal guardians were fully informed about the nature of the study and the benefits of participating. Both parental/caregivers written consent and study participants' assent were obtained before the data collection. For adults, written consent was obtained from both the parents and study participants.

## 4 RESULTS

### 4.1 Sociodemographic characteristics

The mean age of the 443 respondents, attending special needs schools in the Amhara region, was 15.8 years (SD = 3.8) with an age range of 7–30 years. More than half of the participants were males (53.5%), Grade 1 to 4 in their educational level (53.5%). The majority of the study participants had a family monthly income of less than 2500 Ethiopian Birr (79.5%). Moreover, one‐third of the study participants were hearing‐impaired students (33.6%) (Table 1).

**Table 1:** Sociodemographic characteristics of special needs school students in Amhara region, Ethiopia (*n* = 443)

Sociodemographic variables		7–12 years *n* (%)	13–18 years, *n* (%)	Above 18 years, *n* (%)	Total, *n* (%)
Sex	Male	36 (8.1%)	138 (31.2%)	63 (14.2%)	237 (53.5%)
	Female	39 (8.8%)	147 (33.2%)	20 (4.5%)	206 (46.5%)
Residency	Gondar	11 (2.5%)	53 (12.0%)	28 (6.3%)	92 (20.8%)
	Bahir‐dar	22 (5.0%)	110 (24.8%)	12 (2.7%)	144 (32.5%)
	Debre‐Markos	22 (5.0%)	75 (16.9%)	36 (8.1%)	133 (30.0%)
	Dessie	20 (4.5%)	47 (10.6%)	7 (1.6%)	74 (16.7%)
Grade level	Grade 1‐4	68 (15.3%)	147 (33.2%)	21 (4.7%)	236 (53.3%)
	Grade 5‐8	7 (1.6%)	112 (25.3%)	30 (6.8%)	149 (33.6%)
	Grade 9‐12	0 (0.0%)	26 (5.9%)	32 (7.2%)	58 (13.1%)
Religion	Orthodox	47 (10.6%)	192 (43.3%)	70 (15.8%)	309 (69.8%)
	Catholic	15 (3.4%)	41 (9.3%)	7 (1.6%)	63 (14.2%)
	Muslim	11 (2.5%)	46 (10.4%)	5 (1.1%)	62 (14.0%)
	Protestant	2 (0.5%)	6 (1.4%)	1 (0.2%)	9 (2.0%)
Paternal educational status	No formal education	56 (13.4%)	220 (52.6%)	63 (15.1%)	339 (81.1%)
	Primary school	4 (1.0%)	11 (2.6%)	7 (1.7%)	22 (5.3%)
	Secondary school	13 (3.1%)	38 (9.1%)	6 (1.4%)	57 (13.6%)
Maternal educational status	No formal education	58 (13.7%)	239 (56.6%)	73 (17.3%)	370 (87.7%)
	Primary school	4 (0.9%)	13 (3.1%)	4 (0.9%)	21 (5.0%)
	Secondary school	9 (2.1%)	19 (4.5%)	3 (0.7%)	31 (7.3%)
Monthly income	≤2500 ETB	49 (12.6%)	225 (57.7%)	69 (17.7%)	343 (87.9%)
	Above 2500 ETB	13 (3.3%)	26 (6.7%)	8 (2.1%)	47 (12.1%)
Type of disability	Visual	14 (3.2%)	74 (16.7%)	42 (9.5%)	130 (29.3%)
	Hearing	27 (6.1%)	98 (22.1%)	24 (5.4%)	149 (33.6%)
	Intellectual	30 (6.8%)	95 (21.4%)	12 (2.7%)	137 (30.9%)
	Physical	4 (0.9%)	18 (4.1%)	5 (1.1%)	27 (6.1%)

Abbreviation: ETB, Ethiopian Birr.

### 4.2 Dental caries in the primary dentition

The prevalence of dental caries in primary dentition was 23.1% (95% CI: 11.9, 32.1). About 16 (32.3%) students of age 7–12 years and 3 (11.7%) students aged 13–18 years had dental caries. The prevalence of dental caries was high among those who had no tooth brushing experience than those who brush their tooth (38.9% vs. 15.8%). Moreover, students with malocclusion had higher dental caries prevalence than those who had no malocclusion (32.1% vs. 14.9%) (Table 2).

**Table 2:** Prevalence of dental caries among students attending special needs schools in Amhara region, Ethiopia

Predictor variables		Dental caries			
		Permanent teeth		Primary teeth	
		*N* (%)	*p* value	*N* (%)	*p* value
Sex	Male	97 (40.9%)	.781	6 (16.7%)	.343
	Female	87 (42.2%)		10 (25.6%)	
Age	7‐12 years	20 (26.7%)	.016	16 (32.3%)	.001
	13‐18 years	127 (44.6%)		3 (11.7%)	
	Above 18 years	37 (44.6%)		0 (0.0%)	
Grade level	1st to 4th	93 (39.4%)	.007	15 (22.1%)	.633
	5th to 8th	56 (37.6%)		1 (14.3%)	
	9th to 12th	35 (60.3%)		0 (0.0%)	
Family monthly income	≤2500 Ethiopian Birr	143 (41.7%)	.869	14 (28.6%)	.118
	Above 2500 Ethiopian Birr	19 (40.4%)		2 (7.7%)	
Paternal educational status	No formal education	137 (40.4%)	.574	13 (23.2%)	.818
	Primary school	10 (45.5%)		1 (25.0%)	
	High school and above	27 (47.4%)		2 (15.4%)	
Maternal educational status	No formal education	155 (41.9%)	.996	14 (24.1%)	.680
	Primary school	9 (42.9%)		1 (25.0%)	
	High school and above	13 (41.9%)		1 (11.1%)	
Disability type	Visual disability	53 (40.8%)	.623	1 (7.1%)	.306
	Hearing disability	58 (38.9%)		7 (25.9%)	
	Intellectual disability	59 (43.1%)		8 (26.7%)	
	Physical disability	14 (51.9%)		0 (0.0%)	
Carbohydrate food intake	Yes	167 (41.5%)	.992	15 (21.4%)	.940
	No	17 (41.5%)		1 (20.0%)	
Tooth brushing habit	Yes	139 (41.2%)	.826	9 (15.8%)	.037
	No	45 (42.5%)		7 (38.9%)	
Parental support during teeth brushing	Yes	29 (50.0%)	.140	5 (22.7%)	.255
	No	109 (39.5%)		4 (11.4%)	
Oral habits (finger sucking habits….)	Yes	46 (49.5%)	.081	3 (20.0%)	.888
	No	138 (39.4%)		13 (21.7%)	
Comorbidity	Yes	28 (49.1%)	.213	4 (33.3%)	.268
	No	156 (40.4%)		12 (19.0%)	
Medication intake	Yes	28 (53.8%)	.054	4 (36.4%)	.209
	No	155 (39.8%)		12 (19.4%)	
Oral hygiene status	Good	25 (28.7%)	.001	6 (20.7%)	.353
	Fair	54 (36.2%)		3 (13.0%)	
	Poor	105 (50.7%)		7 (30.4%)	
Malocclusion	Yes	87 (47.5%)	.031	9 (32.1%)	.003
	No	97 (37.3%)		7 (14.9%)	

### 4.3 Dental caries in the permanent dentition

The prevalence of dental caries was 41.5% (95% CI: 36.3, 46.0) in permanent dentition. The prevalence of dental caries increased as the age of the study participants increased (*p* = .016). The prevalence was 60.3% among high school students, 39.4% among Grade 1–4 students, and 50.7% among students with poor oral hygiene status. Moreover, students with malocclusion had a higher prevalence of dental caries than those who had no malocclusion (47.5% vs. 37.3%, *p* = .031) (Table 2).

### 4.4 The decayed missed, and filled teeth (DMFT/dmft)

The mean DMFT score of special needs school students was 1.3 ± 1.6. The decayed (D) part took the major component of the DMFT score with a mean value of 1.6 ± 0.5. The mean missed (M) and filled (F) were 0.3 ± 0.8 and 0.1 ± 0.1, respectively. The mean DMFT was high in those above 18 years (DMFT = 1.7 ± 1.9), Grade 9–12 (1.8 ± 1.9), and poor oral hygiene status (1.6 ± 1.9). Moreover, the DMFT score was high in physically disabled students (1.9 ± 1.6). The mean dmft score in the primary dentition was 1.9 ± 0.2. The mean dmft score was 2.0 for 7–12 years old students and visually impaired students (Table 3).

**Table 3:** The DMFT/dmft score among special needs school students in the Amhara region, Ethiopia

Predictor variables		DMFT score	dmft score
		Mean (SD)	Mean (SD)
Sex	Male	1.3 (1.7)	1.9 (0.2)
	Female	1.3 (1.6)	1.9 (0.2)
	Total	1.3 (1.6)	1.9 (0.2)
Age	7–12 years	0.8 (1.7)	2.0 (0.0)
	13–18 years	1.2 (1.5)	1.9 (0.2)
	Above 18 years	1.7 (1.9)	
	Total	1.3 (1.6)	1.9 (0.2)
Grade level	1st to 4th	1.3 (1.7)	1.9 (0.3)
	5th to 8th	1.1 (1.4)	2.0 (0.2)
	9th to 12th	1.8 (1.9)	
	Total	1.3 (1.6)	1.9 (0.2)
Family monthly income (Ethiopian Birr)	≤2500	1.3 (1.6)	1.9 (0.2)
	>2500	1.5 (1.7)	2.0 (0.1)
	Total	1.3 (1.6)	1.9 (0.2)
Malocclusion	Yes	1.5 (1.6)	1.9 (0.3)
	No	1.2 (1.6)	2.0 (0.2)
	Total	1.3 (1.6)	1.9 (0.2)
Disability type	Visual	1.2 (1.6)	2.0 (0.1)
	Hearing	1.2 (1.6)	1.9 (0.2)
	Intellectual	1.3 (1.7)	1.9 (0.3)
	Physical	1.9 (1.6)	1.9 (0.2)
	Total	1.3 (1.6)	1.9 (0.2)
Oral hygiene status	Good	0.9 (1.3)	1.9 (0.2)
	Fair	1.0 (1.2)	2.0 (0.2)
	Poor	1.6 (1.9)	1.9 (0.2)
	Total	1.3 (1.6)	1.9 (0.2)
Medication intake	Yes	1.5 (1.7)	1.8 (0.4)
	No	1.3 (1.6)	1.9 (0.2)
	Total	1.3 (1.6)	1.9 (0.2)
Comorbidity	Yes	1.4 (1.6)	1.8 (0.4)
	No	1.3 (1.6)	1.9 (0.2)
	Total	1.3 (1.6)	1.9 (0.2)
Tooth brushing habits	Yes	1.3 (1.7)	1.9 (0.2)
	No	1.2 (1.5)	1.9 (0.3)
	Total	1.3 (1.6)	1.9 (0.2)
Carbohydrate intake	Yes	1.3 (1.6)	1.9 (0.2)
	No	1.6 (2.1)	1.9 (0.2)
	Total	1.3 (1.6)	1.9 (0.2)

Abbreviations: DMFT, decayed, missed, and filled permanent teeth; dmft, decayed, missed, and filled primary teeth.

### 4.5 Risk indicators of dental caries

As shown in Table 4, age, grade level, oral hygiene status, medication use, malocclusion, comorbidity, and parents' support for teeth brushing and oral habits had a *p*‐value of <0.25 in the bivariable logistic regression model and entered into the multivariable logistic regression model.

**Table 4:** Risk indicators of dental caries in disabled students attending special need schools in Amhara region, Ethiopia

Predictor variables		Dental caries		COR	AOR
		Yes	No		
Age	7–12 years	20 (26.7%)	55 (73.3%)	2.2 (1.1, 4.3)	3.6 (1.6, 8.3)
	13–18 years	127 (44.6%)	158 (55.4%)	1.0 (0.6, 1.6)	1.4 (0.8, 2.5)
	Above 18 years	37 (44.6%)	46 (55.4%)	1	1
Grade level	1st to 4th	93 (39.4%)	143 (60.6%)	2.3 (1.3, 4.2)	2.4 (1.3, 4.4)
	5th to 8th	56 (37.6%)	93 (62.4%)	2.5 (1.4, 4.7)	2.4 (1.3, 4.6)
	9th to 12th	35 (60.3%)	23 (39.7%)	1	1
Comorbidity	Yes	28 (49.1%)	29 (50.9%)	0.7 (0.4, 1.2)	0.9 (0.3,2.8)
	No	156 (40.4%)	230 (59.6%)	1	1
On medication	Yes	28 (53.8%)	24 (46.2%)	0.6 (0.3,1.0)	0.5 (0.2,1.5)
	No	155 (39.8%)	234 (60.2%)	1	1
Oral hygiene status	Good	25 (28.7%)	62 (71.3%)	1	1
	Fair	54 (36.2%)	95 (63.8%)	1.8 (1.2, 2.8)	1.4 (0.8,2.4)
	Poor	105 (50.7%)	102 (49.3%)	2.6 (1.5, 4.4)	2.5 (1.3,4.8)
Malocclusion	Yes	87 (47.5%)	96 (52.5%)	1.5 (1.1, 2.2)	1.5 (0.9, 2.3)
	No	97 (37.3%)	163 (62.7%)	1	1
Parental support during teeth brushing	Yes	29 (50.0%)	29 (50.0%)	1	1
	No	109 (39.5%)	167 (60.5%)	1.5 (0.9, 2.7)	2.2 (1.2, 4.1)
Oral habits	Yes	46 (49.5%)	47 (50.5%)	0.7 (0.4, 1.1)	0.9 (0.5, 1.7)
	No	138 (39.4%)	212 (60.6%)	1	1

Abbreviations: AOR, adjusted odds ratios; COR, Crude odds ratios.

In the multivariable logistic regression analysis, students who were within 7–12 years old were associated with significantly higher odds of dental caries experience than those who were above 18 years old (odds ratio [OR] = 3.6, 95% confidence interval [CI]: 1.6, 8.3).

Factors associated with a significantly higher odds of having dental caries were being graded 1–4 students (AOR: 2.4, 95% CI; 1.3, 4.4), Grade 5–8 (AOR: 2.4, 95% CI: 1.3, 4.6), and having poor oral hygiene status (AOR: 2.5, 95% CI: 1.3, 4.8). Also, students who did not have parental support during tooth brushing had significantly higher odds of having dental caries than those who had parental support during tooth brushing (AOR: 2.2, 95% CI: 1.2, 4.1) (Table 4).

## 5 DISCUSSION

People living with disability have more oral health problems and untreated dental diseases than nondisabled (Lewis et al., 2005; Reid et al., 2003). The present study found that 41.5% of the study participants had dental caries. Age, educational status of the students, oral health status, and lack of parent support during tooth brushing were the predictors of dental caries.

In the present study, 41.5% of students attending special needs schools had dental caries in their permanent dentition, which is consistent with studies done in the United States (45%) (Anderson, 2002), India (38%) (Mehta et al., 2015), Malaysia (54.9%) (Mokhtar et al., 2016), and Rwanda (42.4%) (Uwayezu et al., 2020). However, a higher prevalence was reported in Pakistan (51%) (Azfar et al., 2008), Turkey (86.4%) (Altun et al., 2010), Thailand (53.6%) (Vichayanrat & Kositpumivate, 2014), and China (55.9%) (Wei et al., 2012). This disparity might be explained by the fact that children in developed counties are more frequently exposed to sugary snacks than African children, which is one of the factors contributing to the development of dental caries (Purohit & Singh, 2012). Moreover, it may be that many of these samples were convenient and not representative. In addition, the prevalence of dental caries in primary dentition among the study participants was 21.3%, which is high compared with a study done in India where 15.18% of deaf and mute children attending special schools in Jaipur city had dental caries in their primary dentition (Yadav et al., 2017).

In the current study, the mean DMFT score among the special need schools students was 1.3 ± 1.6, which is consistent with studies done in Malaysia (DMFT; 1.22 ± 2.23) (Mokhtar et al., 2016) and China (DMFT; 1.40 ± 1.89) (Wei et al., 2012) on deaf‐muted high school students. However, the present result is low compared with a study done in Pakistan among special needs children (DMFT;1.85 ± 2.28) (Purohit & Singh, 2012). A higher DMFT score was reported in Thailand among hearing‐impaired students (DMFT of 3.90 ± 3.22) (Vichayanrat & Kositpumivate, 2014). On the other hand, the mean dmft score was 1.95 ± 0.2 in the present study, which was higher than a previous study in India (1.1 ± 2.4) (Duddu et al., 2016). This study found that the decayed component of DMFT was several times greater than that of filled teeth, indicating that parents or caregivers were unconcerned, lacked knowledge, had a negative attitude, or had trouble getting dental health care for their children.

This study found a statistically significant association between age and dental caries. Students of the age group of 7–12 years were associated with significantly higher odds of having dental caries than those who were above 18 years. Similarly, a study done in India reported that individuals between the age of 13–17 years were more likely to have dental caries (Chand et al., 2014). Moreover, a cross‐sectional study done in Rwanda on children living with disabilities revealed that children in the age group of 7–9 years had higher dental caries compared with older age groups (Uwayezu et al., 2020). However, a cross‐sectional study in Thailand found a nonsignificant association between age and dental caries (Vichayanrat & Kositpumivate, 2014). The high prevalence of dental caries in children might be due to children in this age group having a lower capacity to brush their teeth than older children and adults.

A cross‐sectional study done in Rwanda among students with disability reported that students with poor oral hygiene status were six times more likely to have dental caries than students with good oral hygiene (Uwayezu et al., 2020). Moreover, Vichayanrat and Kositpumivate in Thailand found that hearing‐impaired students with poor oral hygiene status were 3.3 times more likely to have dental caries than those with fair or good oral hygiene status (Vichayanrat & Kositpumivate, 2014). Similarly, our study found that students with poor oral hygiene status were 2.5 times more likely to have dental caries compared with those who have good oral hygiene status. The high risk of dental caries among poor oral hygiene students might be due to the high accumulation of Streptococcus mutants and lactobacillus in poor oral hygiene and its effect on the initiation and progression of dental caries.

Previous literature revealed that frequent intake of carbohydrate foods increases the risk of dental caries (Ismail et al., 2008; Jigjid et al., 2009; Niji et al., 2010). However, this study found a nonsignificant association between carbohydrate intake and dental caries, which has a similar finding to a study done in China, where a frequent intake of carbohydrate foods did not influence the prevalence of dental caries (Liu et al., 2014). Furthermore, Rodriguez et al. (2002) reported that carbohydrate intake was not significantly associated with dental caries (Munoz‐Rodrigues et al., 2020) This might be due to the nature of the participants where they are disabled and some of them, especially intellectually disabled individuals, presumably, failed to remember their previous dietary habits (Zahara et al., 2013).

A cross‐sectional study done in Bahir Dar, Ethiopia indicated that students in Grade one to Grade 4 were 3.9 times more likely to have dental caries than their seniors (Mulu et al., 2014). Interestingly, our study also shows a significant association between students' grade level and the prevalence of dental caries. In addition, a cross‐sectional study in Lithuanian on school students revealed that students with a parental educational status of higher educational level had better oral health than those who had a low educational level (Saldūnaitė et al., 2014). A preliminary cross‐sectional study done in India found that students with below‐average academic performance had high caries index (Garg et al., 2012).

People living with disability have a higher prevalence of dental caries than the general population due to their disability, parents' attitudes, and lack of support during tooth brushing (Altun et al., 2010; Anderson, 2002; Mehta et al., 2015). This study found that students who did not have parents' support during tooth brushing were 2.2 times more likely to have dental caries than those who had parental support. Children living with a disability often depend on parents for oral hygiene practices compared with nondisabled children who take care of their oral health (Kadam et al., 2014).

### 5.1 Strength and limitations of the study

The current study is the first comprehensive study on the oral health of the disabled population in Ethiopia. However, we faced some challenges during the study process. First, due to COVID‐19, some special needs school students withdraw from their studies. Second, the study only included students living with disability and attending special needs schools in the region. Third, due to the cross‐sectional nature of the study, a conclusion about the causal relationship between the independent variables and dental caries cannot be drawn. Furthermore, the study only targeted individual‐level factors of dental caries, hence future researchers should focus on factors, such as professionals, infrastructure, and community water fluoridation. Regardless of the above‐listed challenges, parents or caregivers were involved in the interview along with their children, and a multiprofessional team that includes dental professionals, public health experts, and special needs experts was involved in the study.

### 5.2 Conclusion and recommendation

A significant amount of special needs school students in the study had dental caries. Dental caries is significantly associated with age, lower grade level, and poor oral hygiene status. The Ministry of health and ministry of education should implement school oral health programs to improve the oral hygiene practice and knowledge of school children. Moreover, parents, legal guardians, and special needs school teachers should get basic oral health education.

## AUTHOR CONTRIBUTIONS

All authors participated in the conception, design of the study and proposal writing, questionnaire development, and data collection. Simegnew Handebo and Amare Teshome Tefera analyzed the data and drafted the manuscript. Amare Teshome Tefera, Tadesse Awoke Ayele, Abebe Much, Kefyalew Ayalew Getahun, Aynishet Adane, Biruk Girma, and Semira Ali reviewed the drafted manuscript. Amare Teshome Tefera and Simegnew Handebo revised the final manuscript. All authors read and approved the manuscript for submission.

## CONFLICT OF INTEREST

The authors declare no conflict of interest.

## ETHICS STATEMENT

The study tool was reviewed and approved by the University of Gondar institutional review board. Furthermore, study permission was obtained at all city educational bureaus and administers to each special needs school. To guarantee data confidentiality, research volunteers were identified using codes, and unauthorized individuals would not have to access the collected data. All study participants, parents, or legal guardians were fully informed about the nature of the study and the benefits of participating. Before data collection written consent was obtained from parents or legal guardians and students.

## Data Availability

All datasets used for this manuscript will be available upon reasonable request to the corresponding author.
